# Searching for Biomarkers in the Blood of Patients at Risk of Developing Parkinson’s Disease at the Prodromal Stage

**DOI:** 10.3390/ijms24031842

**Published:** 2023-01-17

**Authors:** Elena A. Katunina, Victor Blokhin, Marina R. Nodel, Ekaterina N. Pavlova, Alexander L. Kalinkin, Valerian G. Kucheryanu, Leyla Alekperova, Marianna V. Selikhova, Mikhail Yu. Martynov, Michael V. Ugrumov

**Affiliations:** 1 Department of Neurology, Neurosurgery, and Medical Genetic, Pirogov Russian National Research Medical University, Moscow 117997, Russia; 2 Federal Center for Brain and Neurotechnologies, Moscow 117513, Russia; 3 Koltzov Institute of Developmental Biology of Russian Academy of Sciences, Moscow 119334, Russia; 4 Russian Gerontological Research and Clinical Center, Ministry of Health of Russia, I.M. Sechenov First Moscow State Medical University, Moscow 119435, Russia; 5 Medical Research and Education Center, Lomonosov Moscow State University, Moscow 119991, Russia; 6 Research Institute of General Pathology and Pathophysiology, Moscow 125315, Russia

**Keywords:** Parkinson’s disease, patients, prodromal stage, blood, biomarkers, dopaminergic neurons

## Abstract

Parkinson’s disease (PD) is diagnosed many years after its onset, under a significant degradation of the nigrostriatal dopaminergic system, responsible for the regulation of motor function. This explains the low effectiveness of the treatment of patients. Therefore, one of the highest priorities in neurology is the development of the early (preclinical) diagnosis of PD. The aim of this study was to search for changes in the blood of patients at risk of developing PD, which are considered potential diagnostic biomarkers. Out of 1835 patients, 26 patients were included in the risk group and 20 patients in the control group. The primary criteria for inclusion in a risk group were the impairment of sleep behavior disorder and sense of smell, and the secondary criteria were neurological and mental disorders. In patients at risk and in controls, the composition of plasma and the expression of genes of interest in lymphocytes were assessed by 27 indicators. The main changes that we found in plasma include a decrease in the concentrations of l-3,4-dihydroxyphenylalanine (L-DOPA) and urates, as well as the expressions of some types of microRNA, and an increase in the total oxidative status. In turn, in the lymphocytes of patients at risk, an increase in the expression of the DA D3 receptor gene and the lymphocyte activation gene 3 (*LAG3*), as well as a decrease in the expression of the Protein deglycase DJ-1 gene (*PARK7*), were observed. The blood changes we found in patients at risk are considered candidates for diagnostic biomarkers at the prodromal stage of PD.

## 1. Introduction

Parkinson’s disease (PD) is the second neurodegenerative disease after Alzheimer’s disease in terms of incidence and severity [1]. Current symptomatic therapy with dopaminergic (Dopaminergic) medications does not prevent disease progression and disability in patients [2]. The relatively low effectiveness of the current PD therapy may in part be due to the late diagnosis of PD if made at the time of presentation with Parkinsonian motor symptoms, tremor, and/or akinetic rigidity, and subsequently, the late start of treatment [2]. In fact, the first motor symptoms in PD patients may appear 20–30 years after the disease onset, as a result of a significant degeneration of the nigrostriatal system of the brain, which is the key part in the regulation of motor function. Indeed, by this time, 50–60% of nigral dopaminergic neurons in the substantia nigra are lost, and the concentration of dopamine (DA) in their axonal projections to the striatum decreases by 70–80% [3,4,5].

Given the above specific characteristics of the pathogenesis of PD, the efforts of researchers are focused on the development of early (preclinical) diagnostics that enable subsequent neuroprotective therapy long before the motor symptoms are present.

It is thought that the successful development of the preclinical diagnosis of PD slows down the death of nigrostriatal dopaminergic neurons owing to neuroprotective therapy. Such treatment may result not only in significant prolongation of the preclinical (prodromal) stage of PD, but also in the prolongation of the normal social and physical activity of patients [5].

There are four approaches to the development of an early diagnosis of PD, each with its own advantages and disadvantages [5,6]. The first approach enables us to identify functional insufficiency in the nigrostriatal dopaminergic system by revealing the reduced DA synthesis in the striatum by means of positron emission tomography, which may precede the onset of motor symptoms for several years to come. The main disadvantage of this approach is that it cannot be used for preventive examination of the population due to technical complexity and high cost [7,8].

Other approaches are based on the assumption that PD is associated with not only the degeneration of dopaminergic neurons of the nigrostriatal system, but also with other monoaminergic neurons, predominantly catecholaminergic ones, in other parts of the central and peripheral nervous system [5,9]. In this case, non-motor symptoms (sleep and olfactory disturbances, enteral dysfunction, etc.) may appear years before the manifestation of the motor phenotype [10]. Early non-motor symptoms are considered to be biomarkers of the prodromal stage of PD [11,12,13]. Based on population studies of PD, an international panel of experts has selected clinical criteria for the diagnosis of PD at the prodromal stage [11], which continue to be refined. Parasomnia is considered as the most specific early symptom of PD. This is a behavioral disorder involving rapid eye movement (REM) during sleep [14]. Together with REM sleep behavior disorder (RBD), it is proposed to draw on olfactory impairment, constipation, daytime sleepiness, depression, and a number of other disorders as diagnostic markers of the prodromal stage of PD [14,15]. It is believed that the specificity of most premotor symptoms is low, but their combination may significantly increase the diagnostic value [16,17].

Given that PD is a systemic disease, its development should be accompanied not only with non-motor dysfunctions, but also with the disorder of neurotransmitter metabolism in central and peripheral neurons, as well as in the internal organs innervated by these neurons. This should lead to changes in body fluids, which, like premotor symptoms, are considered as potential diagnostic biomarkers [18,19,20]. Proceeding from this concept, the second most widely used approach to the development of a preclinical diagnosis of PD is based on the search for changes in body fluids, blood, cerebrospinal fluid, and tears in untreated patients at an early clinical stage. These changes are considered as systemic manifestations of neurodegeneration and neuroplasticity, and thus biomarkers of the preclinical stage of PD [5,6]. The main disadvantage of this approach is that where a preclinical diagnosis of PD is not available, researchers look for biomarkers in patients at the clinical stage. Since PD is a progressing disease, the biomarkers at the clinical stage cannot be completely identical to biomarkers of PD at the preclinical stage. Consequently, despite the long-term use of this approach, biomarkers of the preclinical stage of PD have not yet been identified and, for this reason, early diagnostics have not been developed [5,21].

Recently, we have proposed a third, hybrid approach to the development of the preclinical diagnosis of PD. It is based on a search for biomarkers in untreated PD patients at an early clinical stage, and in animal models of the preclinical and early clinical stages of PD [5,20,22]. In such case, only those changes in body fluids that are characteristic of both patients and animal models are considered as biomarkers of the preclinical stage. Our recent comparative analysis of blood and tear fluid biomarkers in untreated patients at the early clinical stage of PD and in animal models of PD have shown that no more than 25% of biomarkers found in patients can be considered as preclinical biomarkers [20,22].

The fourth approach to the development of the preclinical diagnosis of PD, which has been mainly used in the last decade, is based on the search for biomarkers in body fluids in patients at risk of developing PD at the prodromal (preclinical) stage. The risk group includes patients who do not have movement disorders, but show premotor symptoms, such as impaired sense of smell, REM behaviour disorder, depression, and constipation [14]. In rare cases, positron emission tomography has been used to confirm that patients, included in the risk group, have a functional insufficiency of the nigrostriatal dopaminergic system. However, this approach has not yet resulted in the development of early PD diagnosis [5,23].

The purpose of this study was to search for blood biomarkers in patients at risk of developing PD at the prodromal stage. To achieve this aim, we had to meet the following objectives: (i) to select the patients at risk according to a neurological examination and the manifestation of premotor symptoms; to obtain blood samples; (ii) to select patients for the control group according to a neurological examination; to obtain blood samples; (iii) to detect changes in the blood of the patients at risk as opposed to those in the control group.

This study extends and deepens our previous pilot study to search for blood changes in patients at risk of developing PD [24]. Thus, in this study, more patients were included in the risk group and the control group. In addition, the set of blood markers to be assessed has been significantly expanded and more advanced data analysis methods have been used.

## 2. Results

### 2.1. Selection and Examination of Patients at Risk for Developing Parkinson’s Disease at the Prodromal Stage

Out of 1835 patients (age: 55–75) screened during the visit to a neurologist or somnologist, 26 patients (15 men, 11 women, mean age—61.0 ± 8.3 years) were included in the risk group. All included patients showed behavioral disorders during the REM sleep phase with RBD, and the average REM Sleep Behavior Disorder Screening Questionnaire (RBDSQ) score was 6.2 ± 3.3 (Table 1).

Using the Sniffin’ Sticks test, olfaction disorders were found in 16 (62%) out of 26 patients (100%). In this group, patients correctly identified on average 9 odors out of 16 odors. However, the results of the test did not always coincide with the self-assessment of the olfaction by patients. Out of 12 patients with complains of reduced olfaction, in 9 (75%) the olfaction disorder was confirmed by the Sniffin’ Sticks test. Among 14 patients who had no olfaction complains, olfaction dysfunction was identified by Sniffin’ Sticks in 7 (50%). In the control group, 12 out of 20 persons responded positively to the question about olfaction disturbance, but only in 5 of them was olfaction disorder identified by the Sniffin’ Sticks test. The mean score on the olfaction scale in the control group was 14.0 ± 2.5 (Table 1).

Constipation on the Scales for Outcomes in Parkinson’s Disease—Autonomic Dysfunction (SCOPA-AUT) was detected in 14 (53%) patients. The frequency of constipation in the control group did not differ significantly from the risk group—45% vs. 53%, respectively. Similarly, there were no significant differences between the two groups in the score on the SCOPA-AUT scale.

According to the Unified Parkinson’s Disease Rating Scale (UPDRS) motor subscale, mild motor symptoms (>2 points) were found in 12 patients. The mean UPDRS score was 2.25 ± 2.19 points (Table 1).

Changes in the Hospital Anxiety and Depression Scale (HADS) were observed in all patients. Depression was found in 23 patients: 12 patients had subclinical depression, and 11 patients had clinical depression. In total, 25 patients had anxiety disorders: 14 had subclinical anxiety, and 11 had clinical anxiety. The combination of depression and anxiety was found in 20 (77%) patients. In the control group, anxiety/depressive disorders were observed in 95% persons. The mean score on HADS anxiety/depression did not differ between the groups (*p* > 0.05) (Table 1).

According to the Starkstein Apathy Scale (SAS), apathy was detected in 15 (57%) patients at risk and in 8 (40%) in the control group. In the risk group, nine (35%) patients complained of fatigue according to The Fatigue Severity Scale (FSS), and daytime drowsiness was observed in seven (27%) patients. In the control group, five (25%) individuals complained of fatigue and four (20%) of daytime sleepiness. The mean scores on SAS, FSS, and ESS did not differ between the groups (Table 1).

For all patients the mean score on the Montreal Cognitive Assessment (MoCA) scale was 24.3 ± 2.9 points. The mean MoCA score for control group was 27.1 ± 1.9 (*p* < 0.05) (Table 1). Mild cognitive impairment—MCI (MoCA—22.1 ± 2.5)—was found in 13 (50%) patients, and in 4 (15.4%) patients the mean MoCA score was 17.0 ± 1.5. In the control group, MCI was observed in seven (35%) individuals (MoCA—22.7 ± 2.1). The number of individuals with MCI/dementia did not differ between groups: χ^2^ (Yate’s corrected) = 3.05, *p* = 0.081, OR = 3.51 95% CI = 0.88–14.52.

Coffee consumption, smoking, medical history of relatives with PD, and exposure to short-term non-professional contact with pesticides were similar in both groups.

In 85% of patients at risk, in addition to behavioral disorders in the REM phase, more than two additional symptoms were identified. Considering the assessment of apathy, fatigue, daytime sleepiness, and cognitive functions, 100% of patients had various combinations of more than two out of non-motor symptoms and mild motor signs.

**Figure 1:**
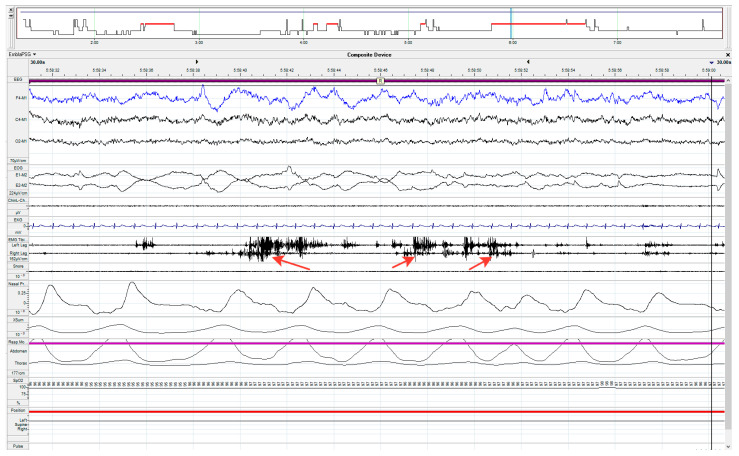
Polysomnography of patient with REM sleep behavior disorder (RBD), 30 s epoch of REM sleep. REM sleep without atonia (RWA). The arrows indicate the transient EMG bursts during REM sleep in the muscle channels (m. tibialis anterior of the left and right legs).

Polysomnography was performed in 14 patients who scored at least 5 points on the RBDSQ scale. In 12 of them, there was no muscle atony during REM sleep, whereas episodes of motor activity were recorded, ranging from individual wince and gesticulation to complex movements such as jumping out of bed (Figure 1). These patients had a mean RBDSQ score of 6.2 ± 3.3 (Table 1). The majority of patients had respiratory problems during sleep, ranging from mild to severe sleep apnea. In two patients, polysomnography showed no abnormalities.

**Figure 2:**
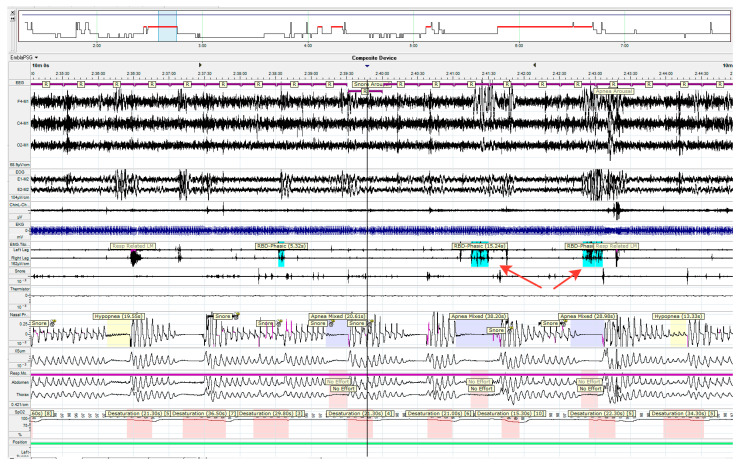
Abnormal motor activity during rapid eye movement sleep during two episodes of mixed apnea (arrows).

In some patients, abnormal motor activity in the REM sleep phase was noted during episodes of apnea, and not at the end of them, as is usually the case for patients with obstructive or central sleep apnea (Figure 2).

There was a positive correlation between anxiety and depression (r = 0.45, *p* = 0.021), depression and apathy (r = 0.42, *p* = 0.033) and RBDSQ and fatigue (r = 0.48, *p* = 0.014), as well as a significant negative correlation between anxiety and increased daytime sleepiness (r = 0.75, *p* = 0.002) and score on MoCA test and increased fatigue (r = 0.54, *p* = 0.005) (Table 2). Other correlations that sis mot proved to be statistically significant presented in Supplementary Table S1.

### 2.2. Biochemical and Molecular-Biological Data on Changes in the Blood of Patients at Risk of Developing Parkinson’s Disease and in Controls

In this study, we have assessed 23 parameters of blood plasma and six parameters of the state of lymphocytes in patients at risk of developing PD and in the control group. However, significant differences between these groups of patients have been found only for eight parameters of plasma and three parameters for the state of lymphocytes. This section of the paper describes only those results that are of particular interest, regardless of whether the corresponding parameters differ in patients at risk and in the control group. Less interesting results are indicated in Supplementary Table S2.

#### 2.2.1. Markers of Oxidative Stress in Plasma

**Figure 3:**
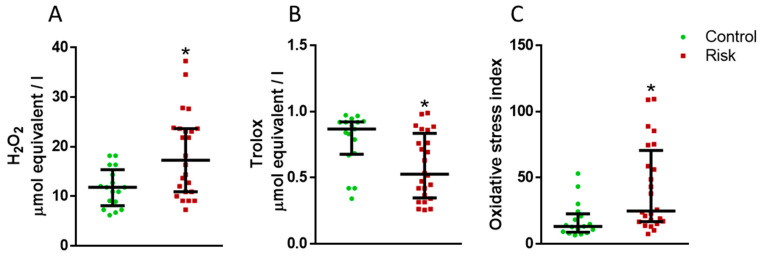
Total oxidant status (**A**), total antioxidant status (**B**), and oxidative stress index (**C**) in the plasma of patients in the control group and in patients at risk of developing Parkinson’s disease. * *p* < 0.05, statistically significant differences. Data presented as a median with an interquartile range and min–max values.

The total oxidant status (TOS) in the plasma of patients at risk of developing PD is increased compared with the control (Figure 3A). On the contrary, the index of total antioxidant status (TAS) in the risk group for developing PD is reduced compared with the control (Figure 3B). The oxidative stress index (OSI) in the plasma of patients at risk was also lower than in the control group (Figure 3C).

#### 2.2.2. microRNA Expression in the Plasma of Patients at Risk of Developing Parkinson’s Disease and in the Controls

**Figure 4:**
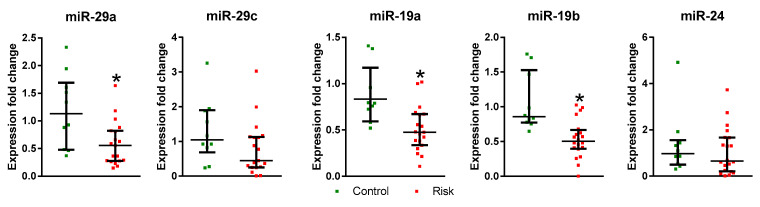
Expressions of miR-29a, miR-29c, miR-19a, miR-19b, and miR-24 in the plasma of patients at risk of developing Parkinson’s disease and in the controls. * *p* < 0.05, statistically significant differences. Data presented as a median with an interquartile range and min–max values.

As a result of our analysis of microRNA expression in plasma, reductions in the expressions of miR-29a, miR-19a, and miR-19b have been found in the patients at risk compared with the control, while the expressions of miR-29c and miR-24 did not change (Figure 4).

#### 2.2.3. Urates in Plasma

**Figure 5:**
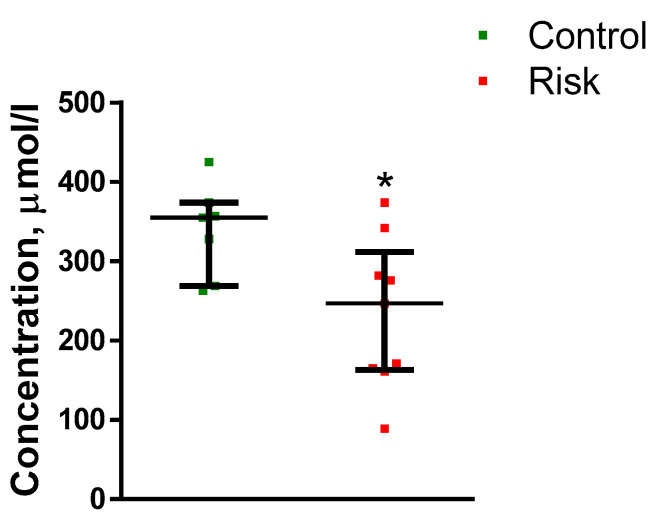
Plasma uric acid concentration in the patients at risk of developing Parkinson’s disease and in the controls. * *p* < 0.05, statistically significant differences. Data presented as a median with an interquartile range and min–max values.

It was shown that the concentration of uric acid in the plasma of patients in the control group was 338.7 µM. In the patients at risk of developing PD, the uric acid concentration decreased to 261.6 µM (Figure 5).

#### 2.2.4. Monoamines and Their Metabolites in Plasma

**Figure 6:**
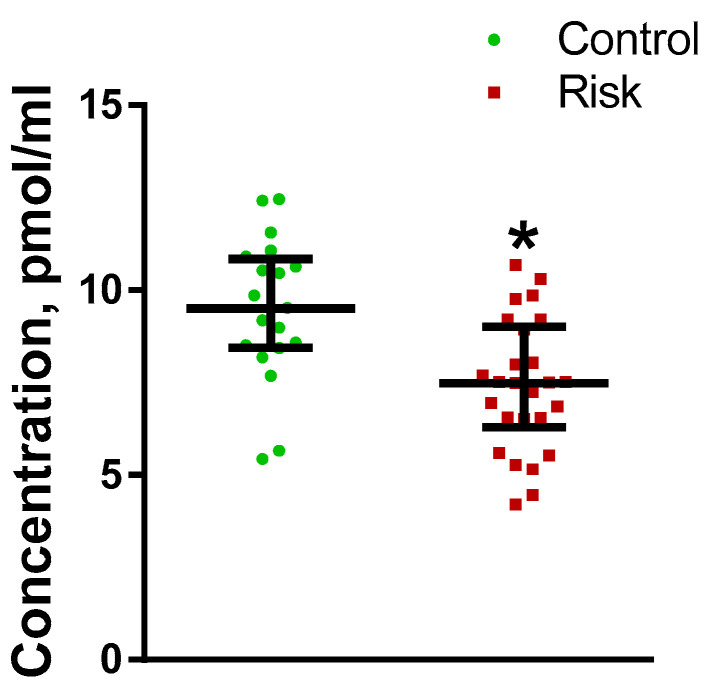
Plasma l-3,4-dihydroxyphenylalanine (L-DOPA) concentration in patients in the control group and in the risk group for developing Parkinson’s disease. * *p* < 0.05, statistically significant differences. Data presented as a median with an interquartile range and min–max values.

Out of all the studied monoamines (norepinephrine, epinephrine, DA, and serotonin) and their metabolites 3,4-dihydroxyphenylacetic acid (DOPAC), homovanillic acid (HVA), 3-o-methyldopa (3-OMD), 3-methoxytyramine (3-MT), and 5-hydroxyindoleacetic acid (5-HIAA), we were only unable to detect DA in plasma. However, the concentrations of norepinephrine, epinephrine, serotonin, DOPAC, HVA, 3-OMD, 3-MT and 5-HIAA in the plasma of patients at risk did not differ from their concentration in the control group. It turned out that only the plasma concentration of l-3,4-dihydroxyphenylalanine (L-DOPA) changed (decreased) in the patients at risk compared with the control (Figure 6).

**Figure 7:**
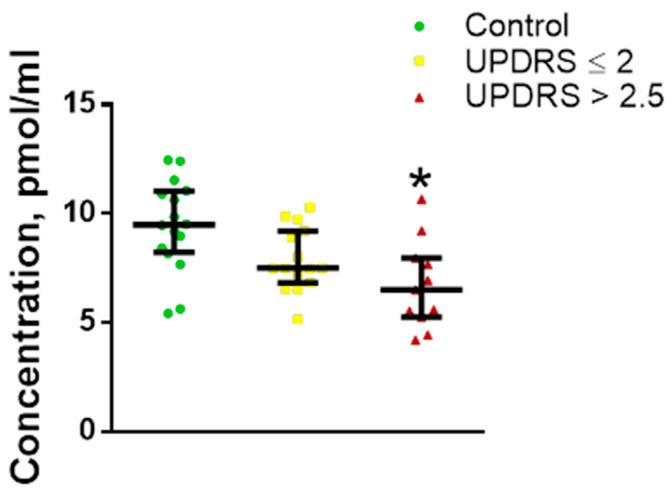
L-3,4-dihydroxyphenylalanine (L-DOPA) concentration in the risk subgroup with mild non-Parkinsonian motor impairment (UPDRS > 2.5 points) and in the risk subgroup without motor impairment (UPDRS ≤ 2 points) compared with control. * *p* < 0.05, statistically significant differences. UPDRS—Unified Parkinson’s Disease Rating Scale. Data presented as a median with an interquartile range and min–max values.

In the risk group, the L-DOPA level in the blood plasma correlated with the results of the Sniffin’ Sticks test (r = 0.55, *p* = 0.012). In addition to assessing the changes in the concentration of L-DOPA in the plasma of patients in the risk group compared with the control, we assessed the plasma L-DOPA concentrations in two selected subgroups. The first subgroup included patients from the risk group with integral UPDRS ≤ 2 points, and the second subgroup included patients with integral UPDRS > 2.5 points. It was shown that the concentration of L-DOPA in the plasma was significantly reduced in the patients of both subgroups (Figure 7).

### 2.3. Quantification of Total, Monomeric, and Oligomeric Plasma α-Synuclein in Patients at Risk for Developing Parkinson’s Disease

**Figure 8:**
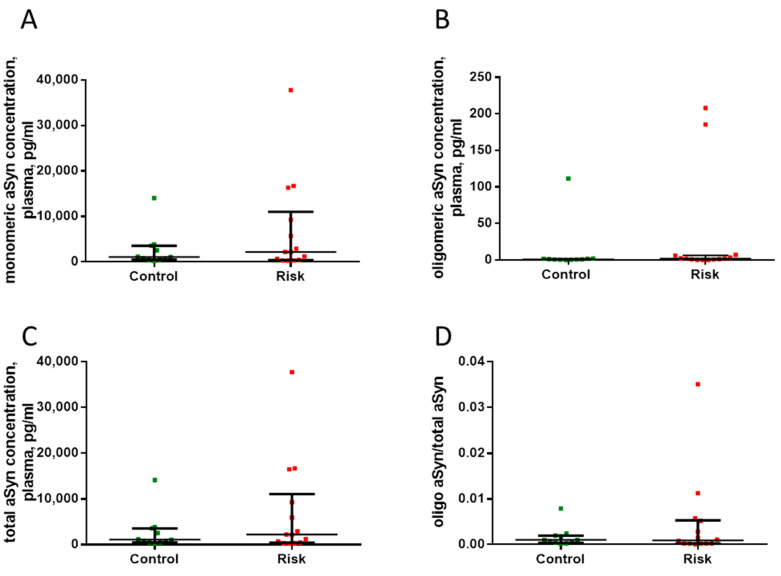
Plasma concentrations of total α-synuclein (**A**), monomeric α-synuclein (**B**), and oligomeric α-synuclein (**C**), as well as the ratio of oligomeric α-synuclein to total α-synuclein (**D**) in the patients at risk of developing Parkinson’s disease and in the controls. Data presented as a median with an interquartile range and min–max values.

We found no differences in the plasma concentrations of total α-synuclein, monomeric α-synuclein, and oligomeric α-synuclein in patients at risk of developing PD and in controls (Figure 8).

### 2.4. Dopamine Receptor Genes in Lymphocytes

**Figure 9:**
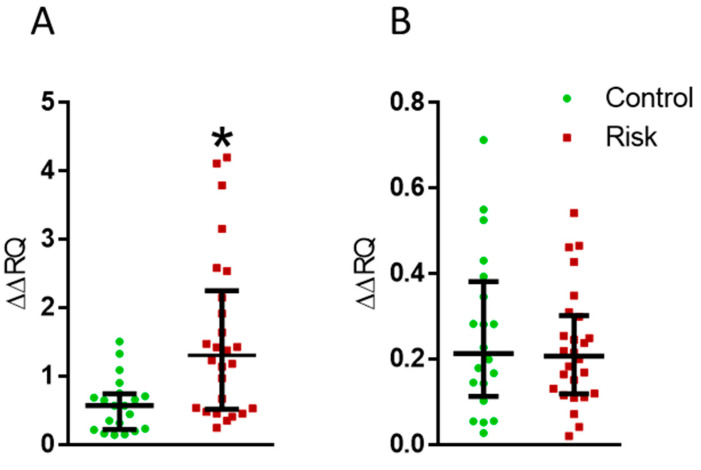
Changes in the expressions of the dopamine D3 (**A**) and D4 (**B**) receptor genes in the patients at risk of developing Parkinson’s disease compared with the controls. ∆∆RQ, the change in the expression of the D3 and D4 receptor genes with normalization to the housekeeping gene. * *p* < 0.05, statistically significant differences. Data presented as a median with an interquartile range and min–max values.

The analysis of the expression of the DA receptor genes D3 and D4 in lymphocytes has shown an increase in the expression of the D3 receptor gene in the patients at risk of developing PD (Figure 9A). However, the expression of the D4 receptor gene in the patients at risk did not change compared with the control (Figure 9B).

### 2.5. Expression of the PARK7 and LAG3 Genes in Lymphocytes

**Figure 10:**
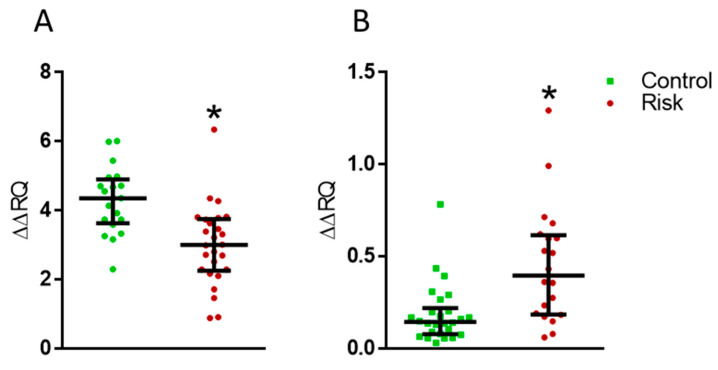
(**A**) Changes in the *PARK7* gene expression in the patients at risk of developing Parkinson’s disease compared with the controls. (**B**) Changes in the *LAG3* gene expression in the patients at risk of developing Parkinson’s disease compared to controls. ∆∆RQ, the change in gene expression normalized to the housekeeping gene. * *p* < 0.05, statistically significant differences. Data presented as a median with an interquartile range and min–max values.

Analysis of the expression of the Protein deglycase DJ-1 (*PARK7*) gene, responsible for the synthesis of the DJ-1 protein, and the lymphocyte activation gene 3 (*LAG3*) receptor gene in lymphocytes has shown a decrease in the expression of the former (Figure 10A) and an increase in the expression of the latter in patients at risk of developing PD, compared with the controls (Figure 10B).

### 2.6. Alpha-Synucleins in Lymphocytes

**Figure 11:**
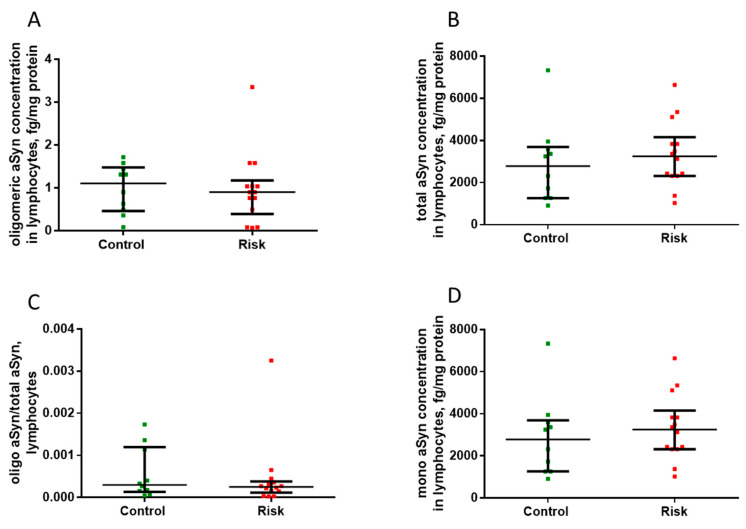
Concentrations of total α-synuclein (**A**), monomeric α-synuclein (**B**), and oligomeric α-synuclein (**C**), as well as the ratio of oligomeric α-synuclein to total α-synuclein (**D**) in lymphocytes in the patients at risk and in the control group. Data presented as a median with an interquartile range and min–max values.

We found no differences in the concentration of total α-synuclein and oligomeric α-synuclein, and we similarly detected no differences in their ratio in the lymphocytes of patients in the risk group and in the control group (Figure 11).

## 3. Discussion

### 3.1. Selection of Patients at Risk for Developing Parkinson’s Disease at the Prodromal Stage: Methodology and Results

Despite the fact that the development of an early (preclinical) diagnosis of PD is one of the highest priorities in neurology, there is still no diagnostic technology recommended for clinical use [5]. An approach based on the identification of premotor symptoms at the prodromal stage of PD, combined with an assessment of changes in body fluids in untreated patients at an early clinical stage of PD, does not allow for diagnosing this disease at the preclinical stage with certainty. Moreover, the biomarkers detected so far are partially specific or non-specific for PD [25].

The efficacy of technologies for the preclinical diagnosis of PD can be essentially improved by searching for biomarkers in body fluids in patients at risk of developing PD at the prodromal stage [26,27]. Using this approach, we performed the screening of 1835 outpatients and selected 26 patients at risk who had non-motor symptoms (sleep disturbance, hypo/anosmia, constipation, anxiety-depressive symptoms), as well as mild motor impairment, insufficient for diagnosing PD. In total, 20 control subjects were also selected out of 1835 outpatients. Control subjects had no RBD and motor symptoms but could have had one of the following symptoms: hyposmia, constipation, and anxiety–depressive symptoms. The main criterion for selecting patients at risk was a behavior disorder in the sleep phase with rapid eye movement on the RBDSQ scale. Sleep disorder is currently considered the most probable marker of the prodromal stage of PD [28]. Approximately 20% of patients note sleep disorders before the onset of motor symptoms [13]. According to prospective studies, 90% of patients with RBD eventually develop synucleinopathy: PD, multiple system atrophy, or diffuse Lewy body disease [29,30]. However, motor symptoms appear several years after the beginning of RBD [29,31]. In 20–40% of patients with RBD, changes in the nigrostriatal dopaminergic system have been detected by DAT-scan [32,33,34]. According to DAT-scan, RBD is a more important symptom of the degeneration of the nigrostriatal dopaminergic system than hyposmia [35]. In our study, somnography was performed in 14 out of 26 patients at risk of developing PD who scored at least 5 points on the RBDSQ. RBD was confirmed in 12 patients. Nevertheless, RBDSQ is considered to be insufficiently sensitive for the preclinical diagnosis of PD. Indeed, 47.4% of de novo patients show false-negative results [36].

Along with changes in RBDSQ, we have selected patients who had at least one additional symptom from the following list: hyposmia, constipation, anxiety–depressive symptoms, and mild motor symptoms. Our analysis showed that 85% of patients at risk had a combination of more than two of these additional symptoms. Considering the additional assessment on the scales of apathy, fatigue, daytime sleepiness, and cognitive functions, all patients included in the risk group had a variety of non-motor symptoms (more than two, on average three to four symptoms per patient) in various combinations, and were associated with mild motor impairment. In our opinion, the presence of the latter significantly increases the risk of developing PD.

When assessing olfaction, hyposmia was revealed in 62% participants at risk of PD. The results of the test did not always coincide with the self-assessment of the olfaction by patients. Out of the 12 patients with complains of reduced olfaction, the olfaction disorder was confirmed in 9 (75%). Among 14 patients who had no olfaction complaints, olfaction disorder was identified in 7 (50%).

We found a high frequency of anxiety–depressive disorders both in the risk group (100%) and in the control group (95%). According to our data, 17 out of 26 (65%) patients in the risk group had MCI, although the mean age of these patients was only 61.0 ± 8.3 years. This is in line with recent studies showing that cognitive dysfunction is one of the earliest manifestations of PD [37].

We found no correlation between RBD and mild motor symptoms. By contrast, there was a positive correlation between RBDSQ scale and fatigue (r = 0.47; *p* = 0.014). Positive correlations were also found between depression and anxiety scores (r = 0.45, *p* = 0.021), as well as depression and apathy (r = 0.42, *p* = 0.033). The MoCA score negatively correlated with fatigue scale (r = −0.54, *p* = 0.005). The lack of significant correlations between (i) RBD and hyposmia and (ii) emotional–affective and cognitive impairments is in line with the concept of different origins of PD, “brain-first” or “body-first” [38,39]. In the brain-first (top-down) subtype, olfactory impairment precedes peripheral autonomic dysfunction. In the brain-first (top-down) subtype, PD begins with impaired sense of smell and then spreads to peripheral autonomic functions [39]. With the body-first (bottom-up) subtype, PD pathology initially arises in the enteric autonomic nervous system, and then spreads along the vagus and sympathetic fibers to the basal ganglia and olfactory system [40]. Premotor RBD is thought to be a marker of the body-first subtype of PD. In this case, the pathological process first reaches the brainstem and then spreads to the substantia nigra [41]. The lack of a significant correlation between RBD symptoms and the presence of constipation observed in our study can be explained by the fact that we assessed enteric function based on three points of the SCOPA-AUT scale. The use of more detailed questionnaires or the assessment of colonic transit time with radioisotope tags could reveal subtle enteric dysfunction [38,42].

In general, PD prodromal biomarkers can be a potential tool for differential diagnosis of PD with other synucleinopathies, in particular multiple sclerosis atrophy (MSA). The differential diagnosis between PD/dementia with Lewy bodies (DLB) and MSA on the clinical ground can represent a significant challenge. At present, there are no definite biological markers for distinguishing the prodromal stage of MSA from the prodromal stage of PD. Recently, the Movement Disorder Society proposed diagnostic criteria aimed at improving the diagnostic accuracy of possible prodromal MSA, but they still require validation in prospective clinical and clinicopathological studies [43]. The possible biomarkers at the prodromal stage of DLB can encompass not only cognitive deficits but also motor signs, sleep disorders, autonomic dysfunction, and neuropsychiatric symptoms. In a prospective study in 88% of those who developed probable DLB, the baseline MCI diagnosis included attention and/or visuospatial deficits. Additionally, those who developed probable DLB were more likely to have baseline daytime sleepiness and subtle Parkinsonism [44]. Besides clinical and cognitive signs, EEG changes and iRBD can also be regarded as possible biomarkers of DLB. A dominant frequency < 8 Hz and dominant frequency variability >1.5 Hz observed on quantitative EEG (QEEG) is typical of DLB, and in a 3-year follow-up study, 83% of subjects with MCI with this pattern on QEEG converted to DLB [45]. iRBD can also be a possible biomarker of the prodromal stage of DLB. In a prospective study [29], 31.8% (14/44) of individuals with iRBD had developed DLB.

RBD has an unquestionable link with α-synucleinopathies [46], although in tauopathies, in particularly progressive supranuclear palsy (PSP), an RBD was found in 25–35% [47,48]. Additionally, sleep architecture may be altered in patients with PSP [49]. Of note, the incidence of tauopathy is significantly smaller than that of PD and DLB, and is reported to be 2.6 per 100,000 for PSP and 0.4 per 100,000 for corticobasal syndrome.

### 3.2. Search for Blood Biomarkers in Patients at Risk of Developing Parkinson’s Disease at the Prodromal Stage: Methodology and Results

**Figure 12:**
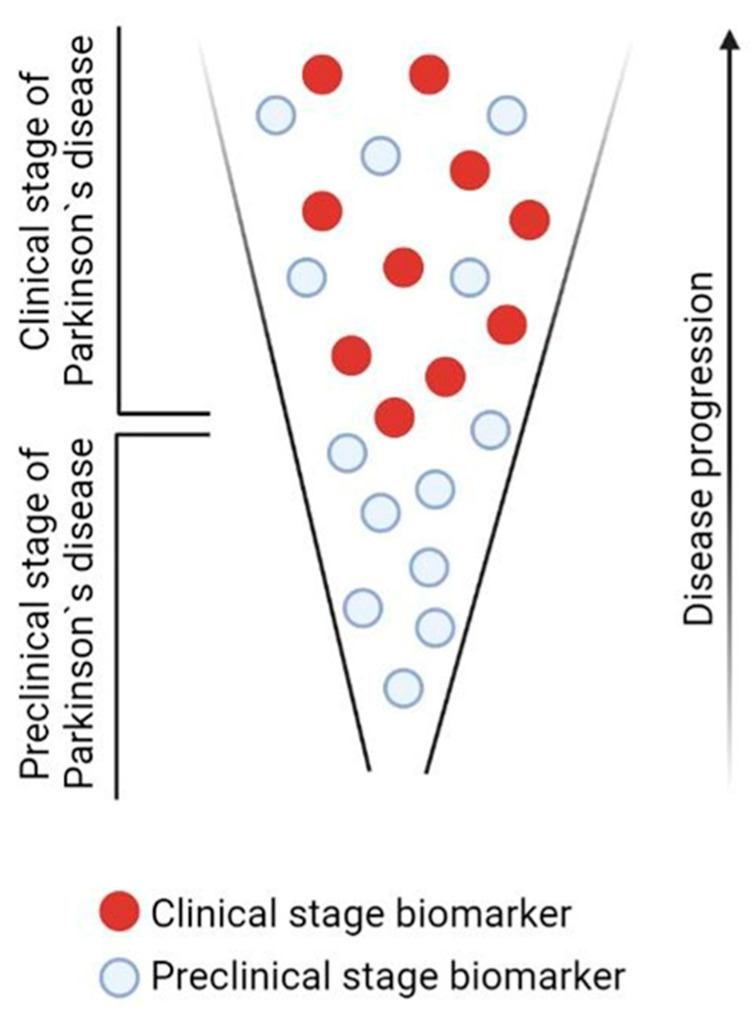
Schematic representation of the emergence of new biomarkers in the blood as Parkinson’s disease progresses.

Given that PD is a systemic disease, the most widely used approach to developing its early diagnosis is based on searching for changes in body fluids, mainly in the cerebrospinal fluid (CSF) and in the blood, in untreated patients at an early clinical stage of PD [27,50]. However, a serious drawback of this approach is that, along with biomarkers of the preclinical stage, biomarkers of the clinical stage of PD are also determined in these patients (Figure 12) [5].

There are a number of approaches to improve the efficiency of the search for diagnostic biomarkers of PD at the preclinical stage. One of them is the search for biomarkers in body fluids in patients at risk of developing PD at the prodromal stage. These patients are selected for the presence of premotor symptoms, in the absence of motor symptoms [51]. Changes in body fluids found in patients at risk of developing PD, in contrast to those detected in untreated patients at an early clinical stage of PD, can be considered with certainty as candidates for diagnostic biomarkers of PD at the preclinical stage. In this study, we assessed changes in 29 blood parameters in patients at risk. They were selected for analysis, taking into account the peculiarities of the pathogenesis of PD and previously obtained data on PD biomarkers found in untreated patients at an early clinical stage of PD [20,22,52,53,54].

#### 3.2.1. Plasma Monoamines and Their Metabolites in Patients at Risk

Since the degeneration of central and peripheral monoaminergic neurons, predominantly catecholaminergic neurons, is a key link in the pathogenesis of PD, the attention of many researchers has been focused on assessing the levels of catecholamines and their metabolites in body fluids, mainly in the CSF and blood plasma. However, the data obtained in these studies are contradictory [55]. Given the progressive development of PD, we hypothesized that only a small fraction of the biomarkers found in body fluids in untreated patients at an early stage of PD could be biomarkers of the preclinical stage [5]. This assumption has been confirmed by comparing our data on changes in plasma levels of catecholamines and their metabolites obtained in our previous study in untreated PD patients at an early clinical stage [20], and in this study in patients at risk of developing PD. Indeed, in untreated patients at an early clinical stage, we found a decrease in the concentration of all studied catecholamines and metabolites, norepinephrine, adrenaline, DA, L-DOPA, and DOPAC in plasma [20], whereas in patients at risk of developing PD, the concentrations of adrenaline, norepinephrine, DA, serotonin, and DOPAC in plasma did not change compared with the control (Table 3).

These differences may be due not only to different degrees of neurodegeneration of catecholaminergic neurons, but also to the differing permeability of the blood–brain barrier. Indeed, the blood–brain barrier, normally impermeable to monoamines, becomes permeable in neurodegenerative diseases [56,57]. The first assumption is more realistic, since we also did not find changes in the concentration of most monoamine metabolites in plasma in patients at risk of PD, although the blood–brain barrier is permeable to them, even in normal cases (Table 3).

Nevertheless, it was logical to assume that the degeneration of dopaminergic neurons in PD and the activation of neuroplasticity, aimed at compensating for DA deficiency in patients at risk, may lead to a change in the concentration of the DA metabolites, primarily L-DOPA and DOPAC, which easily cross the blood–brain barrier. Indeed, we have shown that the concentration of L-DOPA in blood plasma decreases in patients at risk. When patients in the risk group were divided into subgroups according to the UPDRS scale, there was a slight but significant decrease in the concentration of L-DOPA in the group of patients with UPDRS less than 2 points and a more pronounced decrease in the group with UPDRS more than 2 points, which is characterized by mild non-Parkinsonian movement disorders. In addition, we found a correlation between a decrease in the level of L-DOPA and an increase in the incidence of olfactory disorders. A decrease in L-DOPA concentration has also been previously found in the CSF of patients at risk of developing PD [52] and in the plasma of untreated patients at the early clinical stage of PD [20]. The decrease in the concentration of L-DOPA in plasma found in this study in patients at risk of developing PD can be due to the death of nigrostriatal dopaminergic neurons, which leads to a decrease in the synthesis of DA and L-DOPA. This is confirmed by a decrease in the concentration of L-DOPA and DA in the CFS [58], as well as by a decrease in the level of 18F-DOPA incorporation (positron emission tomography) into dopamine synthesis [51] and DAT binding (DAT scan) in nigrostriatal dopaminergic neurons in patients at risk of PD development [59].

A less significant reason for the decrease in the concentration of L-DOPA in the blood may be its increased involvement in the synthesis of DA in dopaminergic neurons, in serotoninergic neurons, and in neurons expressing only aromatic L-amino acid decarboxylase [60,61,62].

Despite our expectations, the plasma concentration of DOPAC did not change in patients at risk compared with the control, although its concentration in plasma was reduced in patients at the clinical stage of PD [20,63]. In contrast to plasma, the concentration of DOPAC decreased in the CSF in patients at the prodromal stage of PD [52,63,64].

#### 3.2.2. Plasma Urates in Patients at Risk of Developing Parkinson’s Disease

In previous studies on the search for blood biomarkers in untreated patients at the early clinical stage of PD, certain changes in body fluids were found, manifesting both systemic neurodegeneration and compensatory processes [5]. One of these biomarkers is urates, which have a neuroprotective effect, preventing the development of oxidative stress [65,66]. Many studies have shown that at the clinical stage of PD, the concentration of urates in the blood is reduced compared to the age control. Moreover, the concentration of urates in the blood gradually decreases as PD progresses [54]. There are only a few studies showing that the plasma urate concentration decreases not only at the clinical stage, but also at the prodromal stage of PD [67,68]. These data are supported by the results of our study. Indeed, we have shown that in patients at risk of developing PD, the plasma urate concentration is reduced by 23% compared with the control group. Plasma urate levels can also be used to assess the risk of developing PD. So, Davis and colleagues showed that among men whose plasma urate levels were above average, the risk of developing idiopathic PD is reduced by 40% [69].

Thus, the change in plasma urate concentration can be considered as one of the diagnostic biomarkers of PD at the prodromal stage, which, however, should be used in combination with other biomarkers.

#### 3.2.3. Plasma Oxidative Stress Index in Patients at Risk of Developing Parkinson’s Disease

Several studies have shown that nonspecific inflammation and oxidative stress can provoke neuronal damage in PD. A recent prospective study found that individuals with gastrointestinal symptoms that required a gastrointestinal endoscopy, but with a finding of histologically normal mucosa or nonspecific inflammation, had an increased subsequent risk of developing Alzheimer’s or Parkinson diseases compared with the population references [70]. In line with this are previously published studies in which lymphocyte count and different patterns of neutrophil-to-lymphocyte or platelet-to-lymphocyte ratio were associated not only with PD, but also with MCI in PD or MSA patients, thus reflecting the inflammatory process in α-synucleinopathies [71,72].

Chronic exposure to exogenous and endogenous toxins can upregulate microglia and lead to the production of reactive oxygen species and proinflammatory cytokines, which cause neurotoxicity and neurodegeneration [73]. In physiological conditions, microglial activation by neuronal α-synuclein results in the formation of autophagosomes for the degradation of α-synuclein via synucleinphagy. The disruption of microglial autophagy promotes the accumulation of misfolded α-synuclein and causes nigral dopaminergic neuron degeneration [74]. Additionally, a recent transcriptomics and functional assessment study revealed that α-synuclein-accumulating microglial cells developed a strong reactive state with phagocytic exhaustion and the excessive production of oxidative and proinflammatory molecules [75].

Metabolic processes in the body are often accompanied by the formation of reactive oxygen species, which leads to oxidative stress. High concentrations of reactive oxygen species disrupt mitochondrial function and lead to cell death [76]. It is interesting that during the synthesis and degradation of DA, a significant amount of reactive oxygen species are formed, which contributes to the death of dopaminergic neurons and the development of PD [77,78]. The body’s inability to inactivate reactive oxygen species is expressed in OSI, which can be considered as one of the biomarkers of neurodegeneration.

According to our data, OSI is elevated in the plasma in patients at risk of developing PD in comparison with the control group. Since OSI is calculated as the ratio of TOS to TAS, it was important to analyze the change in each of these parameters. We have found an increase in TOS and a decrease in TAS in plasma in patients at risk, which is consistent with the results of previous data obtained from patients with diagnosed PD [79,80]. Moreover, our data on changes in ASI in patients at risk of developing PD correlate with our data on changes in the plasma concentration of urates, which are powerful antioxidants [81,82,83,84]. In fact, an increase in OSI in patients at risk of developing PD is accompanied by a decrease in plasma urate concentration. Given that urates have an antioxidant effect, our data on OSI suggest that already at the prodromal stage of PD, the mechanisms to prevent oxidative stress are impaired, which promote the development of PD.

Thus, the change in OSI can be considered as a biomarker of PD at the prodromal stage.

#### 3.2.4. Plasma Synucleins in Patients at Risk of Developing Parkinson’s Disease

One of the most important mechanisms of the pathogenesis of PD is the inhibition of α-synuclein degradation by the ubiquitin–proteasome system in neurons, which is accompanied by the accumulation of α-synuclein and its transformation into oligomeric complexes that are toxic to neurons, especially to dopaminergic neurons of the nigrostriatal system [85,86,87,88]. Previous studies have shown that the concentration of total monomeric α-synuclein decreased in the CSF and plasma of patients with PD, while the concentration of toxic oligomeric α-synuclein increased compared with the control group [58,89,90,91,92,93]. Based on these data, the ratio of the content of oligomeric α-synuclein to the content of monomeric α-synuclein in body fluids is considered to be the most representative diagnostic biomarker of PD [27,90,94].

The above literature data on changes in the metabolism of α-synuclein in nigrostriatal dopaminergic neurons and changes in the content of α-synuclein in the blood prompted us to compare the concentrations of α-synucleins in the blood of patients at risk of developing PD with patients in the control. We have found no differences in plasma concentrations of total α-synuclein, monomeric α-synuclein, and oligomeric α-synuclein in these groups of patients. Our data seem to contradict the current idea that α-synucleinopathy is one of the early manifestations of the pathogenesis of PD [95].

#### 3.2.5. MicroRNAs in Plasma in Patients at Risk of Developing Parkinson’s Disease

When studying the peripheral manifestations of PD in the last decade, much attention has been paid to the content in body fluids of microRNA, short non-coding nucleotide sequences that bind to mRNA causing its degradation. MicroRNAs have been shown to play an important role in the pathogenesis of many diseases, including PD [96,97]. Thus, in patients with PD, an increase in the concentrations of miR-144-5p, miR-542-3p and miR-200a-3p in the CSF and miR-4639-5p in plasma has been found [98,99]. In this study, we attempted to search for changes in microRNAs expression in blood plasma in patients at risk of developing PD. We selected miR-19a, miR-19b, miR-29a, miR-29c, and miR-24 for analysis. Indeed, miR-19a and miR-19b expression correlate with changes in the expression of SRPK2 associated with PD [100,101]. We also chose miR-29a and miR-29c for analysis, since their blood concentration changes in PD, correlating with disease severity [102], as well as miR-24, since it is also associated with PD [103].

Of the five microRNAs studied, the expressions of three of them (19a, 19b, and 29a) decreased in plasma in patients at risk of developing PD, whereas the expressions of the other two microRNAs (24 and 29c) did not change. In the control group, we observed a positive correlation between miRNA-19a expression and L-DOPA levels, as well as between miRNA-29c expression and smell test, while no such correlations were found in the risk group. These data provide indirect evidence of the disintegration at the prodromal stage of PD of physiological processes, which are normally regulated by miRNA-19a and miRNA-29c.

Thus, based on our data, some microRNAs can be considered as candidates for diagnostic biomarkers of PD at the prodromal stage.

#### 3.2.6. Expression of Dopamine Receptor Genes in Lymphocytes in Patients at Risk of Developing Parkinson’s Disease

In addition to searching for changes in plasma in patients at risk of developing Parkinson’s disease, we have also assessed changes in blood cells. In this context, of particular interest are lymphocytes, which have the entire chemical machinery for DA synthesis and express all types of DA receptors [104,105,106]. In addition, it has previously been shown in patients with PD that the expression of genes for some DA receptors is reduced and the expression of genes for other receptors is increased [53,107]. In fact, in untreated patients with PD, lymphocytes showed an increase in the expression of the D3 receptor gene and no change in the expression of the D4 receptor gene [53]. Similarly, we have found an increase in the expression of the D3 receptor gene in patients at risk of developing PD compared with controls. When evaluating the functional significance of the increased expression of the D3 receptor gene in patients at risk of developing PD, one should proceed from the notion that DA, acting on these receptors, activates the anti-inflammatory activity of lymphocytes [108,109].

#### 3.2.7. Changes in the Expression of PARK7 and LAG3 in Lymphocytes in Patients at Risk of Developing Parkinson’s Disease

*PARK7* encodes the DJ-1 protein, which is an important component of the body’s antioxidant system [110]. Mutations in this gene lead to the development of Parkinsonism [111]. We have shown for the first time that *PARK7* expression is reduced in patients at risk of developing PD compared with the controls, which is consistent with the increase in the plasma oxidative stress index described above. Both facts indicate a disruption of the antioxidant system in our risk group patients and can be considered as candidates for diagnostic biomarkers of PD at the prodromal stage.

In addition to assessing the expression of *PARK7* in lymphocytes in patients at risk of developing PD, the analysis of the expression of *LAG3* was of great interest. This interest is explained, on the one hand, by the fact that LAG3 belongs to the immunoglobulin superfamily expressed by peripheral immune cells, microglia, and neurons, and, on the other hand, by the fact that LAG3 interacts with α-synuclein fibrils during neuroinflammation [112,113]. In addition, LAG3 overexpression has been shown to promote α-synuclein phosphorylation at serine-129, which is one of the hallmarks of synucleinopathy in PD [114]. We have shown for the first time that *LAG3* gene expression is increased in patients at risk of developing PD and can be considered as a candidate for diagnostic biomarkers of PD at the prodromal stage.

## 4. Materials and Methods

### 4.1. Patients

The study included 46 patients (27 men, 19 women), with a mean age of 62.5 ± 10.3 years. These patients were selected from 1835 people, aged 55 to 75 years, who, for whatever reason, turned to a neurologist or a somnologist. All patients signed informed consent to participate in this study.

### 4.2. Approaches to the Neurological Examination of Patients

**Figure 13:**
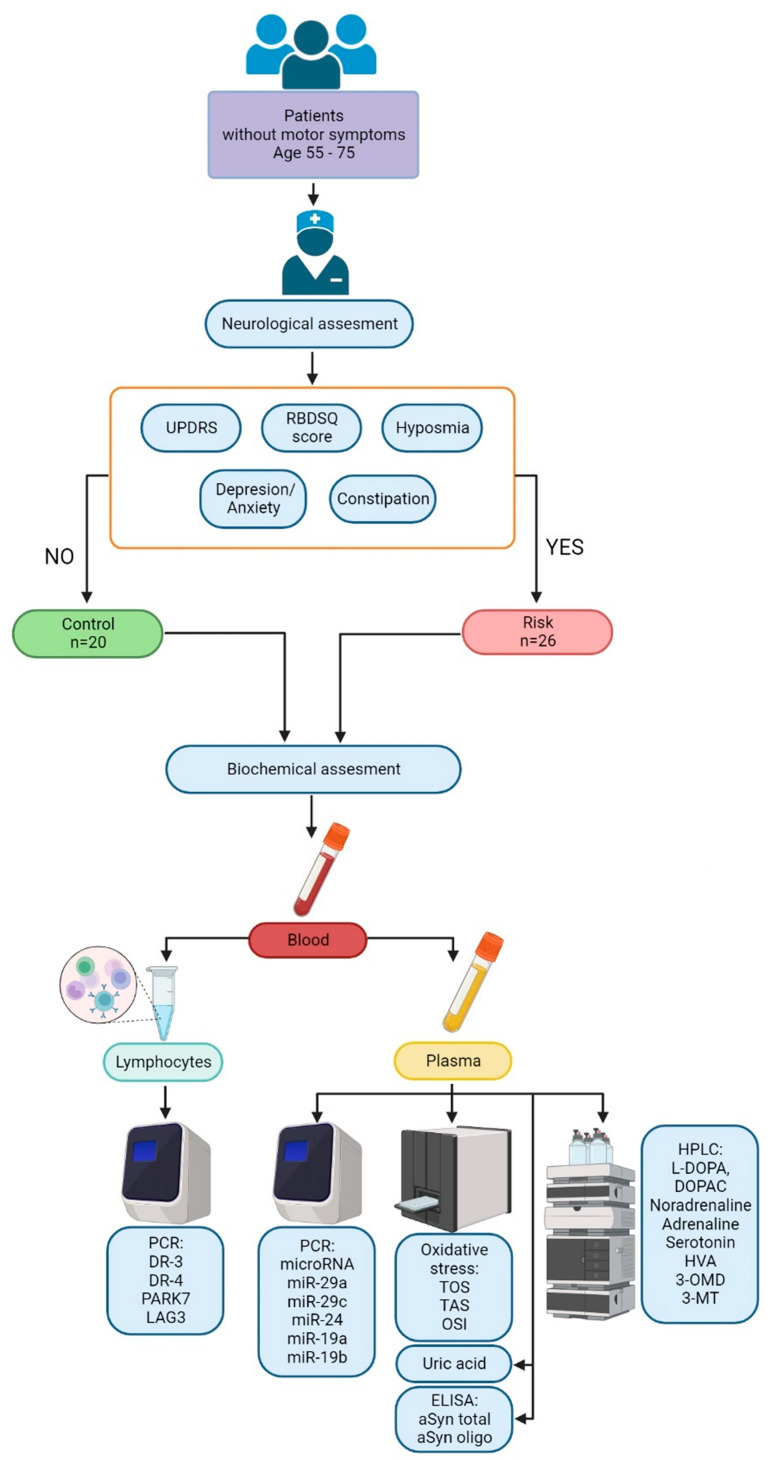
Study design to include patients in the risk group for developing Parkinson’s disease at the prodromal stage and in the control group, as well as to search for changes in the blood considered potential biomarkers of the preclinical stage. RBDSQ—the REM Sleep Behavior Disorder Screening Questionnaire; UPDRS—Unified Parkinson’s Disease Rating Scale, TOS—total oxidant status; TAS—total antioxidant status; OSI—oxidative stress index; aSyn total—total alpha-synuclein; aSyn oligo—oligomeric alpha-synuclein; L-DOPA—l-3,4-dihydroxyphenylalanine; DOPAC—3,4-dihydroxyphenylacetic acid; HVA—homovanillic acid, 3-OMD—3-O-methyldopa; 3-MT—3-methoxytyramine; PARK7—protein deglycase DJ-1; LAG3—lymphocyte-activation gene 3; DR-3—dopamine receptor 3; DR-4—dopaminer receptor 4.

To assess the neurological status of patients, the following approaches have been used: (i) RBDSQ [115] to identify sleep disturbance in the form of motor activity caused by dreams; (ii) assessment of smell using a commercial test system Sniffin’ Sticks (Burghart Medizintechnik, Wedel, Germany), which includes 16 felt-tip markers with different smells; (iii) the SCOPA-AUT patient survey to assess intestinal motility disorders [116]; (iv) the HADS survey of patients to assess anxiety and depression [117]; (v) assessment of motor impairment according to Part III (Motor impairment) of the UPDRS [118]; (vi) the assessment of cognitive functions according to the MoCA scale; (vii) the assessment of apathy according to the SAS [119,120]; (viii) the assessment of fatigue according to the FSS [121], and (ix) the assessment of daytime sleepiness according to the Epworth Sleepiness Scale (ESS) [122] (Figure 13).

### 4.3. Criteria for the Inclusion of Patients in the Risk Group

As candidates for inclusion in the risk group, we considered patients who: (i) had sleep disorder complaints or were informed about sleep disorders by their close relatives and received at least 5 points out of the maximal possible number of 12 points on the RBDSQ; (ii) when determining standard odors using the Sniffin’ Sticks test systems, gave a wrong answer at least 12 times out of the 16 possible options; (iii) had complaints of constipation and responded positively to two out of the three questions on the SCOPA-AUT scale; (iv) scored at least 8 out of 21 on the depression or anxiety subscale of the HADS; (v) scored between 2 and 6 out of 124 on motor symptoms on the UPDRS Part III (Motor Disorders) and met the MDS criteria for prodromal Parkinson’s disease [14].

According to a neurological examination, the risk group included patients who met at least two of the above criteria. The first and obligatory criterion was a sleep behavior disorder on the RBDSQ scale. As additional criteria, hyposmia, constipation, anxiety–depressive symptoms, and mild motor symptoms were used (Table 4). According to the above criteria, the risk group included 26 patients, 15 men and 11 women, mean age 61 ± 8.3 years.

### 4.4. Criteria for the Inclusion of Patients in the Control Group

The control group included patients who did not have RBD and showed no changes on the UPDRS scale. The second requirement for the inclusion of patients in the control group was the presence of no more than one of the following symptoms: hyposmia, constipation, and anxiety/depression. As a result, the control group included 20 patients, 12 men and 8 women, with a mean age 64 ± 6.3 years.

### 4.5. Criteria for the Exclusion of Patients from the Trial

The exclusion criteria from the clinical examination were inflammatory intestinal disease, cancer or intestinal surgery, ENT pathology, psychiatric or neurological brain diseases that could cause hyposmia, constipation, emotional and affective disorders, and cognitive dysfunction.

### 4.6. Additional Examination of Patients at Risk and of Patients in Control Group

#### 4.6.1. Cognitive Function

In patients who had passed the initial selection for the risk or control group, we additionally assessed the cognitive functions according to the MoCA scale, as well as the presence of apathy [119], fatigue according to the FSS [121], and sleepiness on the ESS. The cognitive functions were considered normal with a score of 26 or more on the MoCA scale. Clinically significant apathy was considered with a score of 14 or more points out of 42. Clinically significant fatigue was considered with a score of more than 4 points [123]. Additionally, in patients at risk of PD and in the control group, the presence of population risk factors or anti-risk factors of PD, such as the regular consumption of coffee (more than two cups per day), smoking, the presence of relatives suffering from this disease, and contact with pesticides or herbicides, was considered as moderate at 7–8 points and as abnormal at 9 or more points out of 24.

#### 4.6.2. Polysomnography Study

Attended full-night PSGs synchronized with video (Embla N7000) were performed for the diagnosis of RBD in fourteen patients at risk of RBD according to RBDSQ. All recordings were performed by Embla RemLogic™ Software. Sleep stages and sleep-associated events were manually scored according to the 2012 The American Academy of Sleep Medicine criteria. Manual quantifications of REM sleep without atonia were used.

### 4.7. The Collection and Processing of Blood

Fasting venous blood was collected in the morning from each patient in the risk or control group in 3 vacuum tubes of 8 mL, each containing sodium EDTA (K3 EDTA) (BD Vacuette). The blood was centrifuged at 4000 rpm for 10 min at +4 °C to separate the plasma from the cells. The resulting plasma was frozen in liquid nitrogen and stored at −70 °C until further analysis.

The precipitated blood cells were resuspended in 11 mL of saline (Solofarm, St Petersburg, Russia). In total, 4 mL of Ficoll solution with a density of *p* = 1.07 (PanEco, Moscow, Russia) was poured into sterile test tubes with a volume of 15 mL, and the cells resuspended in saline were layered on it. The tubes were centrifuged at 1500 rpm for 15 min at +4 °C. The lymphocyte fraction was transferred into 15 mL test tubes, and the volume of the solution was adjusted to 15 mL with saline. The tubes were then centrifuged for 10 min at 4000 rpm, and the supernatant was removed. The precipitate was resuspended in 500 µL of 0.02 M phosphate-buffered saline (PBS, pH = 7.3) and transferred to 1.5 mL tubes. The lymphocytes were washed with 1 mL PBS and centrifuged for 5 min at 10,000 rpm. This manipulation was performed twice, and the lymphocyte precipitate was frozen in liquid nitrogen and stored at −70 °C until further analysis of gene expression of *DR-3, DR-4, PARK7,* and *LAG3*.

### 4.8. High-Performance Liquid Chromatography

In total, 50 µL of 1M HClO_4_ and DHBA at a final concentration of 25 pmol/mL were added to thawed plasma samples (500 µL). These samples were centrifuged at 14,000 rpm for 20 min at +4 °C, and the supernatant was taken to measure the contents of monoamines (norepinephrine, epinephrine, and serotonin) and their metabolites (DOPAC, HVA, 3-OMD, 3-MT, and 5-HIAA). Monoamines and metabolites were separated on a column (C18 Reprosil-Pur ODS-3, 3 µm, 100 × 4 mm, Dr. Maisch, Ammerbuch, Germany) at a mobile phase rate of 1.0 mL/min, generated by a pump (LC-20 Adsp, isocratic mode), at a pressure of 140 bar, and at +30 °C (pH = 2.58). The mobile phase included: 0.1 M citrate-phosphate buffer (pH 3.0), 1.1 mM octanesulfonic acid, 0.1 mM EDTA (all reagents from Sigma), and 0.8% acetonitrile (Panreac). A detector (Antec DECADE II) with a glassy carbon cell (+0.85 V), a 50 µm spacer, and an Ag/AgCl reference electrode filled with saturated KCl, was used for the measurement. The peaks of monoamines and metabolites were identified by the release time of the standard solution. The content of monoamines was calculated by the internal standard method as the ratio of peak areas in a mixture of standards of measured monoamines and their metabolites and in plasma using the LabSolutions software (Shimadzu).

### 4.9. Determination of the Oxidative Stress Index

To determine the oxidative stress index (OSI), the following indicators of oxidative stress were measured: total oxidative status (TOS) and total antioxidant status (TAS). The OSI was calculated as the ratio of TOS (µmol H_2_O_2_ Eq/L) to TAS (mmol Trolox Eq/L) [79,124].

TOS was assessed in plasma using the colorimetric method [125]. For this, 105 µL of plasma was added to 675 µL of reagent 1, followed by 33 µL of reagent 2. Reagent 1 includes 150 µM xylenol orange, 140 mM NaCl, and 1.35 M glycerol in 25 mM H_2_SO_4_, pH 1.75, and reagent 2 includes 5 mM Fe(NH_4_)_2_(SO_4_)∙26H_2_O and 10 mM o-Dianisidine, dissolved in 25 mM H_2_SO_4_. Optical density was measured on a spectrophotometer (Hitachi 320, Tokyo, Japan) twice: after mixing reagent 1 and plasma at a wavelength of 800 nm (background) and 4 min after adding reagent 2 at a wavelength of 560 nm. A calibration curve was built with H_2_O_2_ and expressed as H_2_O_2_ micromolar equivalent per liter (µmol H_2_O_2_ Eq/l).

Plasma TAS was also assessed using the colorimetric method. To 600 µL of reagent 3 we added 15 µL of plasma and then 30 µL of reagent 4. Reagent 3 includes o-dianisidine (10 mM), iron ions Fe(NH_4_)2(SO_4_)2 × 6H_2_O 45 µM in Clark and Lubs solution (75 mM, pH = 1.8), and reagent 4 consists of 7.5 mM H_2_O_2_ in Clark and Lubs solution. Optical density was measured on a spectrophotometer (Hitachi 320, Japan) at a wavelength of 444 nm twice, after mixing reagent 3 and plasma (background) and 4 min after adding reagent 4. A calibration curve was constructed with the antioxidant Trolox (6-hydroxy-2,5,7,8-tetramethylchroman-2-carboxylic acid), a water-soluble analog of vitamin E. The results were expressed in Trolox millimolar equivalent per liter (mmol Trolox Eq/L).

### 4.10. Measurement of Urates in Plasma

Plasma uric acid concentration was measured by the enzymatic colorimetric method at a wavelength of 546 nm using an Advia 1800 biochemical analyzer (Siemens Healthcare Diagnostics, Erlangen, Germany).

### 4.11. Isolation of Total RNA from Lymphocytes

In total, 1 mL of TRI-reagent (Sigma, Saint Louis, MO, USA) was added to thawed lymphocytes for RNA extraction, and they were incubated for 5 min at room temperature. The samples were then homogenized by pipetting. The resulting homogenate was incubated for another 5 min at room temperature. Then, 100 μL of 1-bromo-3-chloropropane (Thermo Fisher Scientific, Waltham, MA, USA) was added to each sample, and the samples were incubated for 15 min at room temperature, vortexing every 5 min. The samples were then centrifuged at 15,000 rpm for 15 min at +4 °C, after which the aqueous phase containing RNA was gently removed and transferred into sterile 1.5 mL tubes free of DNase and RNase. For better RNA precipitation, the samples were supplied with isopropanol in a volume equal to the aqueous phase (on average 450–500 µL) and 1 µg of glycogen (Thermo Fisher Scientific, USA). The tubes were kept on a vortex for 5 min at room temperature and were then centrifuged at 15,000 rpm for 10 min at +4 °C, followed by collection of the supernatant. In total, 1 mL of 80% ethanol was added to the resulting precipitate, followed by its stirring, centrifugation at 15,000 rpm for 10 min at +4 °C, and washing with 80% ethanol. The supernatant was collected, and the tubes were left open to allow the remaining ethanol to evaporate. The dry residue was dissolved in 20 µL of DNase and RNase free water. The RNA concentration in the resulting solution was measured using a NanoDrop 8000 spectrophotometer (Thermo Fisher Scientific, USA).

### 4.12. cDNA Synthesis

For cDNA synthesis, 1 μg of RNA was treated with DNase according to the manufacturer’s instructions. Then, 1 µL of EDTA was added to this sample, followed by its incubation for 10 min at +65 °C and RNA precipitation by adding 1 μL of 8 M sodium acetate and 100 μL of 96% ethanol. Finally, everything was thoroughly mixed on a vortex and left overnight at −20 °C. The supernatant was then removed, leaving a precipitate. The test tubes with the precipitate were left with open lids for 15 min to allow the remaining ethanol to evaporate. The precipitate was then dissolved in 11.5 µL of water, and 1 µL of random hexamer primers was added. The tubes were incubated for 5 min at +65 °C, and 7.5 µL of the mixture from the RevertAid H Minus First Strand cDNA synthesis kit (Thermo Fisher Scientific, USA) was added to them.

### 4.13. PCR in Real Time

The resulting cDNA was used for quantitative PCR analysis of the expression of DA receptor genes D3 and D4 receptors, *PARK7* and *LAG3* in lymphocytes (Table 5).

For real-time PCR, the commercial mixture qPCRmix-HS SYBR and LowRox (Evrogen, Moscow, Russia) containing polymerase, buffer, and a mixture of nucleotides was used in accordance with the manufacturer’s manual. In total, 500 ng of the synthesized cDNA was taken into the reaction, followed by the addition of 1 µL of a mixture of forward and reverse primers at a final concentration of 1 µM and 5 µL of the qPCR mix-HS SYBR and LowRox mixture. This mixture was adjusted with water to a volume of 25 µL. The PCR of each gene was repeated three times. Changes in receptor gene expression were assessed by the ΔΔCt method:

∆∆Ct = ∆Ct(Sample) − ∆Ct(Control average).

### 4.14. Isolation of microRNA from Plasma and cDNA Synthesis

In total, 5 µL of 200 nM ath-mir159a was added as an internal standard (ThermoFischer) to 250 μL of thawed plasma samples. MicroRNAs were isolated from plasma using the miRNeasy Serum/Plasma Kit (Qiagen, Hilden, Germany) according to the manufacturer’s protocol. The resulting microRNA samples were frozen and stored at −70 °C until further analysis. The TaqMan Advanced miRNA cDNA synthesis kit (ThermoFischer) was used to synthesize cDNA from miRNAs according to the manufacturer’s protocol. In total, 2 µL of microRNA isolated from plasma was taken for reverse transcription. The resulting cDNA samples were frozen and stored at −70 °C until further use.

### 4.15. PCR of microRNA

Real-time PCR was used to assess changes in the levels of hsa-miR29c, hsa-miR29a, hsa-miR19b, hsa-miR19b, and hsa-miR24 in plasma. For PCR, cDNA was diluted 10 times and 5 µL of diluted cDNA was taken for each reaction. For this reaction, commercial primers TaqMan Advanced miRNA Assay and PCR mixture 2× Fast Advanced master Mix were used according to the manufacturer’s protocol. Real-time PCR was performed three times using a QuantStudio 12K Flex system (Applied Biosystems, Waltham, MA, USA). Changes in miRNA expression were assessed by the ΔΔCt method.

**Measurement of α-synuclein and its derivatives in plasma and lymphocytes**

The concentration of α-synuclein in plasma and in lymphocytes was measured using ELISA kits. Total alpha-synuclein was measured using the hSyn total ELISA kit (Roboscreen, Leipzig, Germany), while for measuring monomeric and oligomeric alpha-synuclein, we used the human a-synuclein patho ELISA kit (Roboscreen, Germany).

**Statistical analysis**

The statistical treatment of clinical data was carried out using the computer program IBM SPSS Statistics 21, version 23.2015. Data are presented as mean ± SD. The correlation between variables depending on the distribution was assessed using the Pearson or Spearman correlation coefficient; 2 × 2 contingency tables with Yate’s correction were employed for comparing of 2 or more frequencies. Differences were considered significant at *p* < 0.05.

The statistical processing of biochemical and molecular biological results was carried out using the statistical package GraphPad Prism Version 6.0 (GraphPad Software, La Jolla, CA, USA).

The data are presented as a median with an interquartile range and min–max values. For the two-group comparison, a nonparametric Mann–Whitney U-test was used. For multiple comparisons, the nonparametric Kruskal–Wallis test was used. In all tests, *p*-values were two-tailed, and *p* < 0.05 was considered statistically significant.

## 5. Conclusions

The tackling of socially significant neurodegenerative diseases, such as Alzheimer’s disease and Parkinson’s disease, is considered one of the global challenges of the 21st century. These diseases remain incurable, and huge financial resources are invested in palliative treatment and the rehabilitation of patients. The main reason for the ineffective treatment of these patients is the late diagnosis and start of treatment of patients. Therefore, the most reasonable way to battle neurodegenerative diseases is the development of early diagnostics—long before the manifestation of clinical symptoms.

This, in turn, will enable us to carry out preventive neuroprotective therapy, which is supposed to slow down the death of neurons and thereby prolong the preclinical stage of a patient’s comfortable life. This study aimed at the development of early (preclinical) diagnosis of PD based on the search for changes in the blood in patients at risk of developing PD at the preclinical (prodromal) stage. To this end, we were assessing the changes in the composition of blood plasma and the functional state of lymphocytes in patients at risk compared with patients in the control group. Patients for these groups were selected according to the presence or absence of pre-motor symptoms compatible with the prodromal stage (impaired RBD and olfaction, constipation, and impaired psycho-emotional status), respectively. The main changes that we found in plasma include a decrease in the concentration of L-DOPA, urates, and in the expressions of some types of microRNA, and an increase in the total oxidative status. At the same time, lymphocytes from patients at risk showed an increase in the expression of genes for the DA receptor D3 and *LAG3*, and a decrease in the expression of *PARK7*.

Thus, the blood changes we found in patients at risk are considered as potential diagnostic biomarkers at the prodromal stage of PD.

This study will be continued and expanded by: (i) increasing the cohorts of patients at risk of developing PD at the prodromal stage and age-matched control group; (ii) expanding the range of analyzed changes in body fluids—not only in the blood, but also in the tear fluid; (c) using positron emission tomography and/or DAT scanning to monitor the state of the nigrostriatal dopaminergic system in patients at risk of developing PD.

## Figures and Tables

**Table 1:** Clinical characteristics of the patients included in the study.

Clinical Scales	Control Group *n* = 20 (12 Men, 8 Women)	Risk Group *n* = 26 (15 Men, 11 Women)
Age, years	64.1 ± 6.3	61.0 ± 8.3
RBDSQ score	2.0 ± 0.5	6.2 ± 3.3 *
SST	14.0 ± 2.5	9.1 ± 4.5 *
SCOPA-AUT	1.05 ± 1.3	1.65 ± 1.20
UPDRS (motor, III part)	0	2.25 ± 2.19 *
HADS anxiety HADS depression	11.6 ± 2.8 9.0 ± 3.2	10.3 ± 2.6 9.8 ± 2.2
SAS	12.5 ± 4.3	14.2 ± 6.3
FSS	3.1 ± 2.3	4.3 ± 2.4
MoCA	27.1 ± 1.9	24.3 ± 2.9 *
ESS	6.5 ± 1.5	7.1 ± 3.9

ESS—the Epworth Sleepiness Scale; FSS—Fatigue Severity Scale; HADS—the Hospital Anxiety and Depression Scale; MoCA—Montreal Cognitive Assessment; RBDSQ—the REM Sleep Behavior Disorder Screening Questionnaire; SCOPA-AUT—Scales for Outcomes in Parkinson’s Disease—Autonomic Dysfunction; SST—Sniffin’ Sticks Test; SAS—Starkstein Apathy Scale; UPDRS—Unified Parkinson’s Disease Rating Scale. *—*p* < 0.05, significant difference. Data presented as mean ± SD.

**Table 2:** Statistically significant correlations between variables.

Variables	RBDSQ	SST	SCOPA-AUT	UPDRS	HADS Anxiety	HADS Depression	SAS	FSS	MoCA	ESS
**RBDSQ**								0.476 *p* = 0.014		
**HADS Anxiety**				−0.388 *p* = 0.050		0.449 *p* = 0.021				−0.749 *p* = 0.002
**HADS Depression**					0.449 *p* = 0.021		0.420 *p* = 0.033			
**SAS**						0.4201 *p* = 0.033				
**FSS**	0.476 *p* = 0.014								−0.544 *p*= 0.005	
**MoCA**								−0.543 *p* = 0.005		
**ESS**					−0.749 *p* = 0.002					

ESS—the Epworth Sleepiness Scale; FSS—Fatigue Severity Scale; HADS—the Hospital Anxiety and Depression Scale; MoCA—Montreal Cognitive Assessment; RBDSQ—the REM Sleep Behavior Disorder Screening Questionnaire; SCOPA-AUT—Scales for Outcomes in Parkinson’s Disease—Autonomic Dysfunction; SST—Sniffin’ Sticks Test; SAS—Starkstein Apathy Scale; UPDRS—Unified Parkinson’s Disease Rating Scale.

**Table 3:** Change in the concentration of monoamines and metabolites in patients with the risk of developing PD compared with control.

Parameter	Patients	
	UPDRS ≤ 2	UPDRS > 2.5
Norepinephrine	→	→
Epinephrine	→	→
L-DOPA	↓	↓
Dopamine	→	→
DOPAC	→	→
5-HTP	→	→
HIAA	→	→
3-MT	→	→
HVA	→	→
3-OMD	→	→
Serotonin	→	→

↓—decrease, → no change. UPDRS—Unified Parkinson’s Disease Rating Scale; L-DOPA—L-3,4-dihydroxyphenylalanine; DOPAC—3,4-dihydroxyphenylacetic acid; 5-HIAA—5-hydroxyindoleacetic acid; 3-MT—3-methoxytyramine; 3-OMD—3-o-methyldopa; HVA—homovanillic acid; 5-HTP—5-hydroxytryptophan.

**Table 4:** Criteria for the inclusion of patients in the risk group and in the control group.

Clinical Scales	Risk Group	Control Group
RBDSQ	≥5	<5
UPDRS	≥2, ≤6	0
SST	≤12	>12
SCOPA-AUT	>1	≤1
HADS		
Anxiety	≥8	<8
Depression	≥8	<8

HADS—the Hospital Anxiety and Depression Scale; RBDSQ—the REM Sleep Behavior Disorder Screening Questionnaire; SCOPA-AUT—Scales for Outcomes in Parkinson’s Disease—Autonomic Dysfunction; SST—Sniffin’ Sticks Test; UPDRS—Unified Parkinson’s Disease Rating Scale.

**Table 5:** Oligonucleotide primers used to assess the expressions of dopamine receptor genes D3 and D4, as well the genes of PARK7 and LAG3.

Gene	Forward Primers	Reverse Primers
*D3*	GTACAGCCAGCATCCTTAATCTCT	ACAGAAGAGGGCAGGACACA
*D4*	GACGCCCTTCTTCGTGGT	GACAGTGTAGATGACGGGGTTG
*PARK7*	GCTGGCGTGCGTTCATT	ACCGTCTCCATTTCCTCTGC
*B2M*	GGGTTTCATCCATCCGACATTG	ACACGGCAGGCATACTCATCTTTT
*LAG3*	TCACAGTGACTCCCAAATCCT	GCTCCACACAAAGCGTTCTT

## Data Availability

Data available on request.

## Supplementary Materials

The following are available online at https://www.mdpi.com/article/10.3390/ijms24031842/s1.

## Abbreviations

CSF—cerebrospinal fluid; DA—dopamine; DAergic—dopaminergic; DOPAC—4-dihydroxyphenylacetic acid; DLB—dementia with Lewy bodies, ESS—The Epworth Sleepiness Scale; FSS—Fatigue Severity Scale; HADS—The Hospital Anxiety and Depression Scale; 5-HIAA—5-hydroxyindoleacetic acid; 5-HTP—5-hydroxytryptophan; HVA—homovanillic acid; LAG3—lymphocyte activation gene 3; L-DOPA—l-3,4-dihydroxyphenylalanine; MCI—mild cognitive impairment; MoCA—Montreal Cognitive Assessment scale; MSA—multiple sclerosis atrophy, 3-MT—3-methoxytyramine; 3-OMD—3-o-methyldopa; OSI—oxidative stress index; PARK7—protein deglycase DJ-1 gene; PD—Parkinson’s disease; PSP—progressive supranuclear palsy, RBD—REM Sleep Behavior Disorder; RBDSQ—REM Sleep Behavior Disorder Screening Questionnaire; REM—rapid eye movement; SAS—Starkstein Apathy Scale; SCOPA-AUT—Scales for Outcomes in Parkinson’s Disease—Autonomic Dysfunction; TAS—total antioxidant status; TOS—total oxidant status; UPDRS—Unified Parkinson’s Disease Rating Scale.
