# Supplementary material for: Searching for Biomarkers in the Blood of Patients at Risk of Developing Parkinson’s Disease at the Prodromal Stage
Source: Int J Mol Sci. 2023 Jan 17;24(3):1842. doi: 10.3390/ijms24031842 (PMC9915927; doi:10.3390/ijms24031842)
Supplement: Supplementary file 1 [file ijms-24-01842-s001.zip › ijms-2121955-supplementary.pdf]

Table S1. Insignificant correlations in the risc group of patients.

	RBDSQ	Odor identification score	SCOPA-AUT	UPDRS	Anxiety (HADS)	Depression (HADS)	Starkstein Apathy Scale (SAS)	Fatigue Severity Scale	MoCA	The Epworth Sleepiness Scale
RBDSQ		-0.2695 P= 0.183	-0.1051 P= 0.609	-0.1804 P= 0.378	0.0699 P= 0.734	-0.0663 P= 0.748	0.0723 P= 0.726		-0.1493 P= 0.476	-0.0847 P= 0.774
SST	-0.2695 P= 0.183		0.0026 P= 0.990	-0.2338 P= 0.250	-0.1453 P= 0.479	0.0329 P= 0.873	-0.3589 P= 0.072	-0.1984 P= 0.331	0.2914 P= 0.158	0.1007 P= 0.732
SCOPA-AUT	-0.1051 P= 0.609	0.0026 P= 0.990		0.2551 P= 0.208	-0.2196 P= 0.281	-0.2658 P= 0.189	0.0443 P= 0.830	-0.1922 P= 0.347	0.0904 P= 0.668	0.3567 P= 0.211
UPDRS	-0.1804 P= 0.378	-0.2338 P= 0.250	0.2551 P= 0.208		-0.3881 P= 0.050	-0.1045 P= 0.612	0.3741 P= 0.060	0.2727 P= 0.178	-0.0406 P= 0.847	0.3224 P= 0.261
Anxiety (HADS)	0.0699 P= 0.734	-0.1453 P= 0.479	-0.2196 P= 0.281	-0.3881 P= 0.050			r-0.0667 P= 0.746	0.0818 P= 0.691	0.1033 P= 0.623	
Depression (HADS)	-0.0663 P= 0.748	0.0329 P= 0.873	-0.2658 P= 0.189	-0.1045 P= 0.612				0.0635 P= 0.758	-0.0411 P= 0.845	-0.1288 P= 0.661
(SAS)	0.0723 P= 0.726	-0.3589 P= 0.072	0.0443 P= 0.830	0.3741 P= 0.060	r-0.0667 P= 0.746			0.2575 P= 0.204	-0.2054 P= 0.325	0.2105 P= 0.470
FSS		-0.1984 P= 0.331	-0.1922 P= 0.347	0.2727 P= 0.178	0.0818 P= 0.691	0.0635 P= 0.758	0.2575 P= 0.204			-0.0399 P= 0.892
MoCA	-0.1493 P= 0.476	0.2914 P= 0.158	0.0904 P= 0.668	-0.0406 P= 0.847	0.1033 P= 0.623	-0.0411 P= 0.845	-0.2054 P= 0.325			-0.1483 P= 0.613
ESS	-0.0847 P= 0.774	0.1007 P= 0.732	0.3567 P= 0.211	0.3224 P= 0.261		-0.1288 P= 0.661	0.2105 P= 0.470	-0.0399 P= 0.892	-0.1483 P= 0.613	

ESS - the Epworth Sleepiness Scale; FSS - Fatigue Severity Scale; HADS - the Hospital Anxiety and Depression Scale; MoCA - Montreal Cognitive Assessment; RBDSQ - the REM sleep behavior disorder screening questionnaire; SCOPA-AUT - Scales for Outcomes in Parkinson’s Disease - Autonomic Dysfunction; SST - Sniffin'Sticks Test; SAS - Starkstein Apathy Scale; UPDRS - Unified Parkinson's Disease Rating Scale.

Table S2. Concentrations of monoamines and some metabolites in plasma of patients in control group and in risk group for developing Parkinson`s disease.

Substance \ Concentration, pmol/ml	Control group	Risk group	P
NA	2.08±0.25	1.84±0.28	0.3390
DOPAC	9.51±1.26	10.97±1.99	0.6883
3-OMD	91.95±12.23	93.9±10.29	0.9803
5-HIAA	41.1±5.51	51.62±5.78	0.3629
5-HTP	2.67±0.83	8.92±2.44	0.0914
HVA	119.34±23.37	101.27±11.08	0.7068
3-MT	69.74±11.08	65.34±14.78	0.6412
5-HT	22.73±7.28	20.31±3.78	0.9773

DA - dopamine; DOPAC - 3,4-Dihydroxyphenylacetic acid; HVA - homovanillic acid; 5-HIAA - 5-Hydroxyindoleacetic acid - 5-Hydroxyindoleacetic acid; 5-HTP - 5-Hydroxytryptophan; 5-HT - 5-hydroxytryptamine (serotonin); 3-MT - 3-Methoxytyramine; 3-OMD - 3-O-Methyldopa; NA - noradrenaline;
$p > 0.05$, non-significant differences
